# A Novel System for the Efficient Generation of Antibodies Following Immunization of Unique Knockout Mouse Strains

**DOI:** 10.1371/journal.pone.0012892

**Published:** 2010-09-23

**Authors:** Anna Hrabovska, Véronique Bernard, Eric Krejci

**Affiliations:** Centre d'Etude de la Sensori-Motricité (CESeM), Université Paris Descartes - CNRS- UMR8194, Paris, France; University of Miami, United States of America

## Abstract

**Background:**

We wished to develop alternate production strategies to generate antibodies against traditionally problematic antigens. As a model we chose butyrylcholinesterase (BChE), involved in termination of cholinergic signaling, and widely considered as a poor immunogen.

**Methodology/Principal Findings:**

Jettisoning traditional laborious *in silico* searching methods to define putative epitopes, we simply immunized available BChE knock-out mice with full-length recombinant BChE protein (having been produced for crystallographic analysis). Immunization with BChE, in practically any form (recombinant human or mouse BChE, BChE purified from human serum, native or denatured), resulted in strong immune responses. Native BChE produced antibodies that favored ELISA and immunostaining detection. Denatured and reduced BChE were more selective for antibodies specific in Western blots. Two especially sensitive monoclonal antibodies were found capable of detecting 0.25 ng of BChE within one min by ELISA. One is specific for human BChE; the other cross-reacts with mouse and rat BChE. Immunization of wild-type mice served as negative controls.

**Conclusions/Significance:**

We examined a simple, fast, and highly efficient strategy to produce antibodies by mining two expanding databases: namely those of knock-out mice and 3D crystallographic protein-structure analysis. We conclude that the immunization of knock-out mice should be a strategy of choice for antibody production.

## Introduction

Monoclonal and polyclonal antibodies are essential tools for biological research. A necessity for structure function studies of proteins both *in vivo* and *in vitro*, antibodies with new and varied properties are constantly in demand. Complications in antibody generation, however, often leave this tool void in the repertoire of those available for the study of many proteins. A commonly held explanation for the failure of antibody production following immunization is a limited antigenic response due to high conservation between the antigen and the endogenous proteins of the immunized host. To circumvent this problem an antigen is designed *in silico* that carries one specific epitope. This strategy involves several steps, including: 1) selection of a primary sequence that is divergent between the different species (immunizing antigen and host to be immunized); 2) evaluation of sequence accessibility in the 3D structure, if available (i.e., presence on the surface of the protein); 3) peptide synthesis and attempts to obtain folding into the native 3D structure and 4) immunization of the distant species. This commonly used strategy can be efficient, despite its complexity and time-consumption. Often, however, non-selectivity (or cross-reactivity) of the antibody is encountered and this problem is usually only uncovered when the antibody is used in a background in which the gene encoding the protein of original interest has been knocked-out or knocked-down [1], [2]. Presence of non-specific labeling or binding in this case is due to the presence of the epitope in other proteins. In cases where the protein of interest is studied in a species in which the deletion of the gene is not possible, the control for cross-reactivity is more difficult.

**Figure 1:**
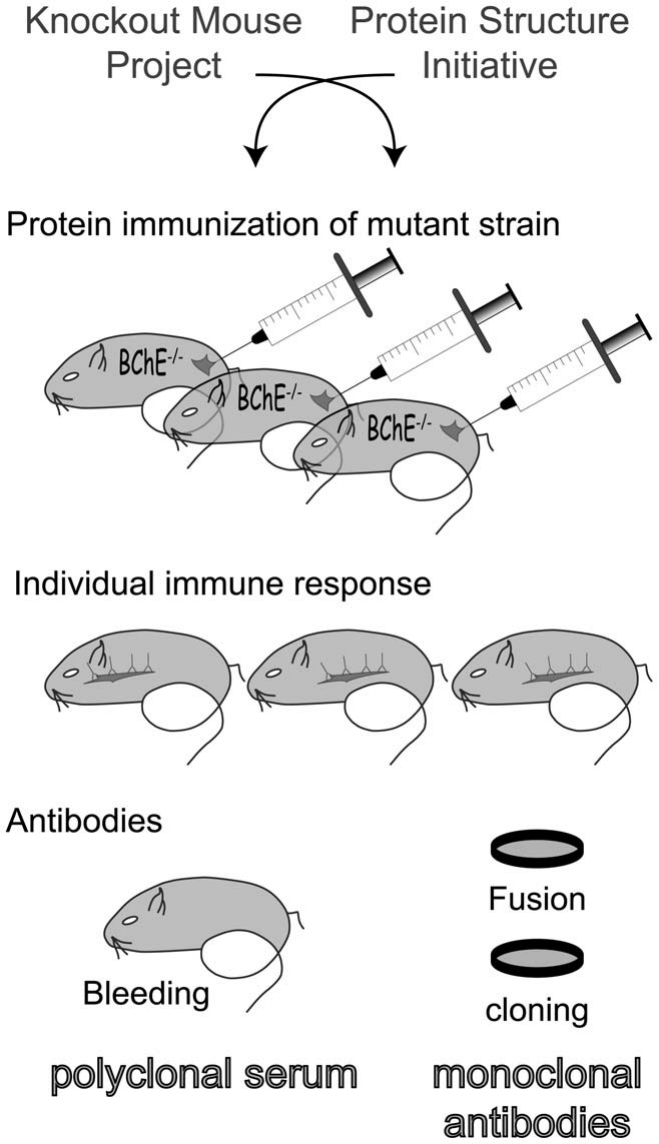
Different steps in the generation of antibodies: Strategy of immunization. Two high throughput methods “Knockout Mouse Project” and “Protein Structure Initiative” are crossed to generate antibodies: immunization of knockout mice with high quality protein domains. Each immunized mouse offers a new collection of antibodies that are used as polyclonal source or that are cloned as monoclonal source after fusion.

In some gene therapy paradigms, on the other hand, unwanted production of an antibody against a selected protein has been described. In these cases an immune-naive host eliminates the newly synthesized protein by standard immune responses, essentially “sabotaging” the gene therapy goal [3], [4]. Along this line, the idea of the immunization of “knock-out” mice was proposed to overcome the problem of inter-species sequence similarity in antibody production [5]. Indeed, this strategy has been successfully used in a few studies but has, however, never become a common method of choice for antibody production. Most likely this is due to the limited variety of genetically modified animals, as well as the lack of a sufficient amount of pure cognate protein for immunization. Whatever the case, here we revisit this issue and shed new light on this simple and efficient mouse immunization strategy (figure 1).

As a test case of this strategy to obtain antibodies, we choose a “problematic” antigen - butyrylcholinesterase (BChE). BChE is a well-characterized enzyme, highly abundant in serum and in tissue, and involved in the hydrolysis of acetylcholine and detoxification of several drugs [6]. During the 1980s, several monoclonal antibodies against human BChE were obtained, but due to their weak affinity they have proved to be not very useful [7], [8]. Currently there are no antibodies either polyclonal or monoclonal that recognize mouse or rat BChE in histochemistry, immunoprecipitation or Western blots. Explanations for this could be that BChE is highly glycosylated and/or the high inter-species conservation of the sequence. For our test of this method we used mice with a complete deletion of the BChE catalytic domain [9]. These animals are without any obvious phenotypic changes. As an immunogen, we first used sugar-diminished full-length recombinant human BChE that was prepared previously to study the 3D structure [10]. In next steps enzyme from different source was used, recombinant mouse BChE or serum human BChE and the antigen was differently prepared (native or denatured).

## Results

### Immunization with recombinant low-sugar protein

**Figure 2:**
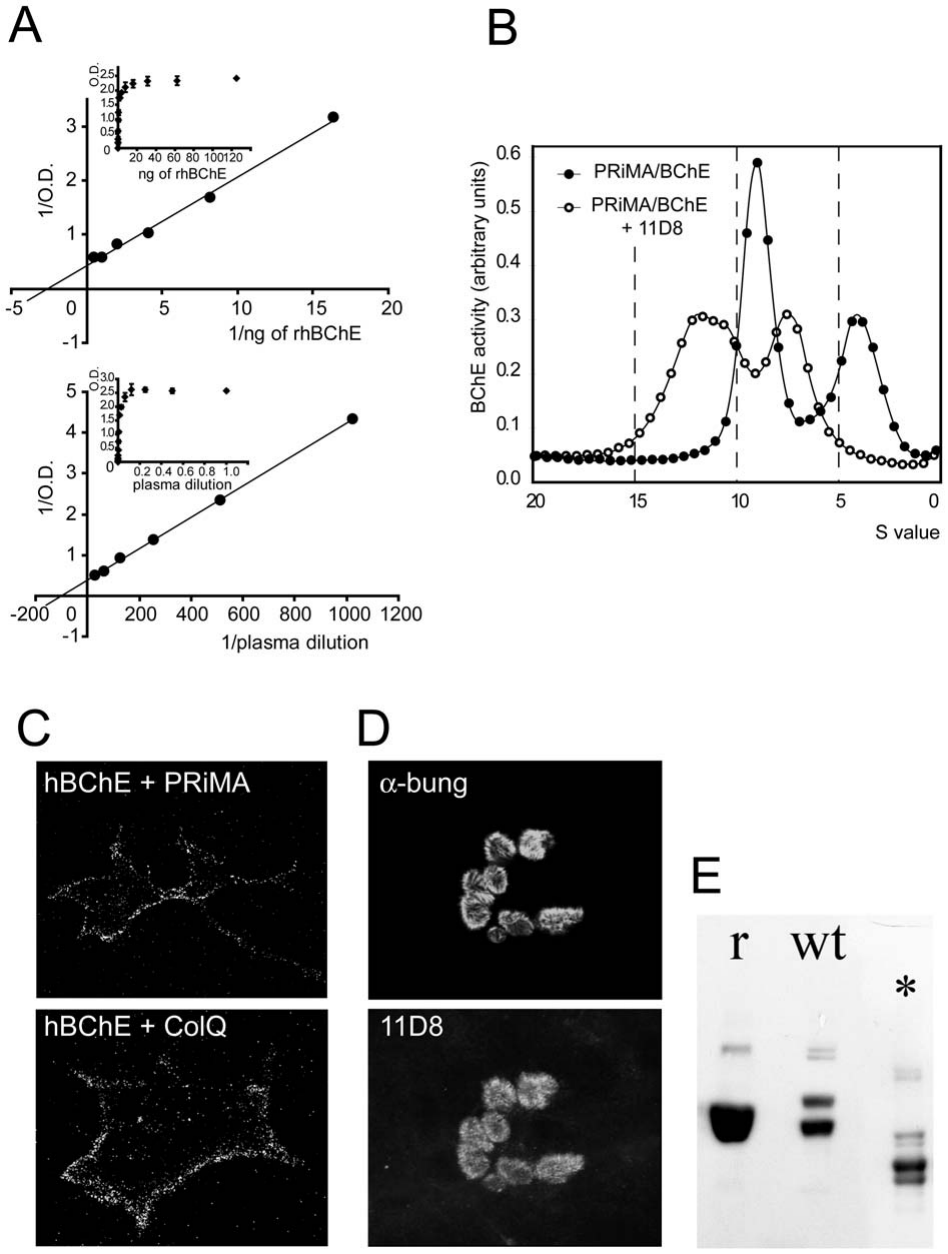
Human BChE is recognized by monoclonal antibody 11D8. (A) Selectivity of 11D8 for recombinant and wild-type BChE by ELISA (60 min detection). (B) 11D8 shifts BChE in sucrose gradient. Sucrose gradient of human PRiMA-BChE complex extracted from COS cells. Binding of 11D8 shifts the peaks of tetramer and dimmer. Wider high-S peak suggests a mixed population of IgG/one tetramer and IgG/two tetramers. (C) 11D8 recognizes BChE expressed by COS cells, anchored by ColQ and PRiMA as documented by confocal microscopy. (D) Immunohistochemistry of human neuromuscular junction (NMJ). Top, nicotinic acetylcholine receptors are visualized with bungarotoxin (Bung). Bottom, staining with 11D8 labels BChE. (E) Western blot of human recombinant (r) and wild-type (wt) BChE, (*) after deglycosylation.

The immune response to the recombinant BChE was strong in all immunized BChE −/− animals as tested in both ELISA and immunohistochemistry of fixed COS cells expressing human BChE. The amount of antibody produced varied from mouse to mouse and did not depend on the amount of injected protein. Even the lowest injected amount of protein (15 µg) gave the maximal respond. As determined in ELISA, after 4 boost injections the antibody titer of the sera with the strongest response was 1/50,000. Wild-type animals, in comparison to BChE −/− mice, responded negligibly to the recombinant BChE and thus were omitted from the study after the first priming injection. One of the BChE −/− mice was used to generate monoclonal antibodies. Out of one thousand hybridomas screened, 33 were positive with recombinant human BChE, and after two further subcultures one IgG1, 11D8, was finally selected. As illustrated in figure 2a and supplementary data (figure S1), 11D8 detected human BChE in ELISA. Within 1 min, 0.25 ng of recombinant protein and 4 nl of human plasma are detected while sensitivity of the detection increases with time; ie. within 1 hour (figure 2a), 0.2 nl of serum and 0.42 pg of rBChE are detected. 11D8 shifted BChE oligomers in sucrose gradient (figure 2b). 11D8 binds fixed human BChE in transfected mammalian cells (figure 2c) and human tissue sections (figure 2d). 11D8 also recognizes purified recombinant and wild-type human BChE in Western blots (figure 2e). Presumably the epitope does not contain glycans because deglycosylated protein in situ is still detected in WB (figure 2e). In addition, the epitope is still present when the different glycosylation sites are deleted from the complete protein (table 1). It is possible, therefore, that the immunization was efficient due to a combination of lowered glycosylation of the recombinant BChE used as an antigen and/or of the use of the BChE knockout strain as host.

**Table 1:** ELISA of extracts from COS cells expressing different mutants of BChE with lowered glycosylation.

h BChE	Glycosylation sites										activity	ELISA
position	17	57	106	241	256	341	455	481	485	486	O.D.	O.D.
4 sugar off		x	x	x	x	x			x		0.62±0.01	1.79±0.03
3 sugar off	x	x	x	x		x			x		1.63±0.01	3.02±0.07
5 sugar off	x	x	x	x					x		1.63±0.11	2.99±0.11
7 sugar off					x	x			x		0.06±0.00	0.14±0.02
6 sugar off		x			x	x			x		0.48±0.01	1.33±0.03
no DNA											0.03±0.00	0.06±0.00
human serum	x	x	x	x	x	x	x	x		x	out of range	out of range

“no DNA” is a COS cell extract of non-transfected cells; 100 µl of rat serum was used as a negative control; 8 µl of human serum was used as a positive control.

### Immunization with BChE purified from human serum

**Figure 3:**
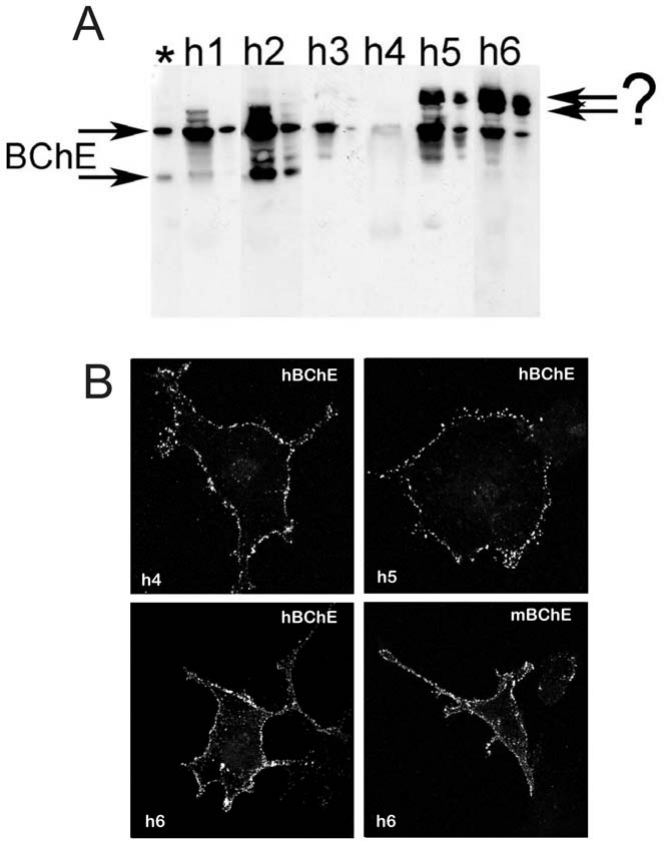
Mouse polyclonal anti-human BChE antibody raised against wild-type protein recognizes human and mouse BChE. (A) Western blot with sera from mice immunized with denatured/reduced protein (h1–h3) or with native protein (h4–h6). (B) Immunohistochemical staining of BChE expressed in COS cells with sera from mice immunized with the native protein (h4–h6); note that h6 recognizes human and mouse BChE. Human sera, 2 µl (first lane) and 0.2 µl (second lane) were loaded on gel; (?) unknown protein; (*) purified wild-type BChE (8 ng).

**Figure 4:**
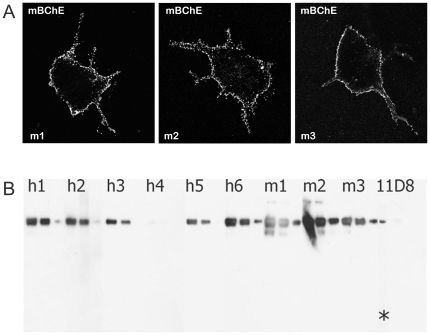
Antibodies from BChE knockout mice immunized with mouse BChE. (A) Confocal microscopy of COS cells expressing mouse BChE. (B) Western blot of recombinant mouse BChE with sera from mice immunized with recombinant mouse BChE (m1–m3), or from mice immunized with BChE purified from human serum (h1–h6), or with monoclonal antibody produced against human low-glycosylated BChE (11D8). In this experiment, each antibody sample was tested with 75 ng, 38 ng and 8 ng of recombinant mouse BChE loaded onto a denaturing gel. In the 11D8 lane, the band marked with “*” represents 750 ng of protein.

To distinguish between these two possibilities, we immunized the BChE knockout strain with wild-type human BChE purified from serum [11]. Mice were immunized in 2 different conditions - with native or denatured/reduced BChE. Similar to the results with recombinant BChE, the immune response to purified human BChE was strong, and detected even after the BChE priming injection. As expected, serum from mice immunized with denatured BChE gave stronger and more selective signal by Western blot (figure 3A) than serum immunized with native enzyme. Sera recognized with different intensity as little as 0.2 µl of human plasma and 8 ng of purified protein. On the other hand, as shown in figure 3B with staining of BChE expressed in a cell line, sera from mice immunized with native BChE better recognized the native enzyme. Interestingly sera from all mice immunized with human BChE cross-reacted with mouse BChE in Western blots (figure 4B) and one serum from immunization with native human BChE, labeled mouse BChE expressed in COS cells (figure 3B). We can conclude, therefore, that anti-BChE antibody production is as efficient regardless of whether the BChE immunogen is glycosylated or not, and that using a mouse that is genetically null for BChE as the target host facilitates previously unobtainable results of anti-BChE antibody production.

### Autologous immunization

**Figure 5:**
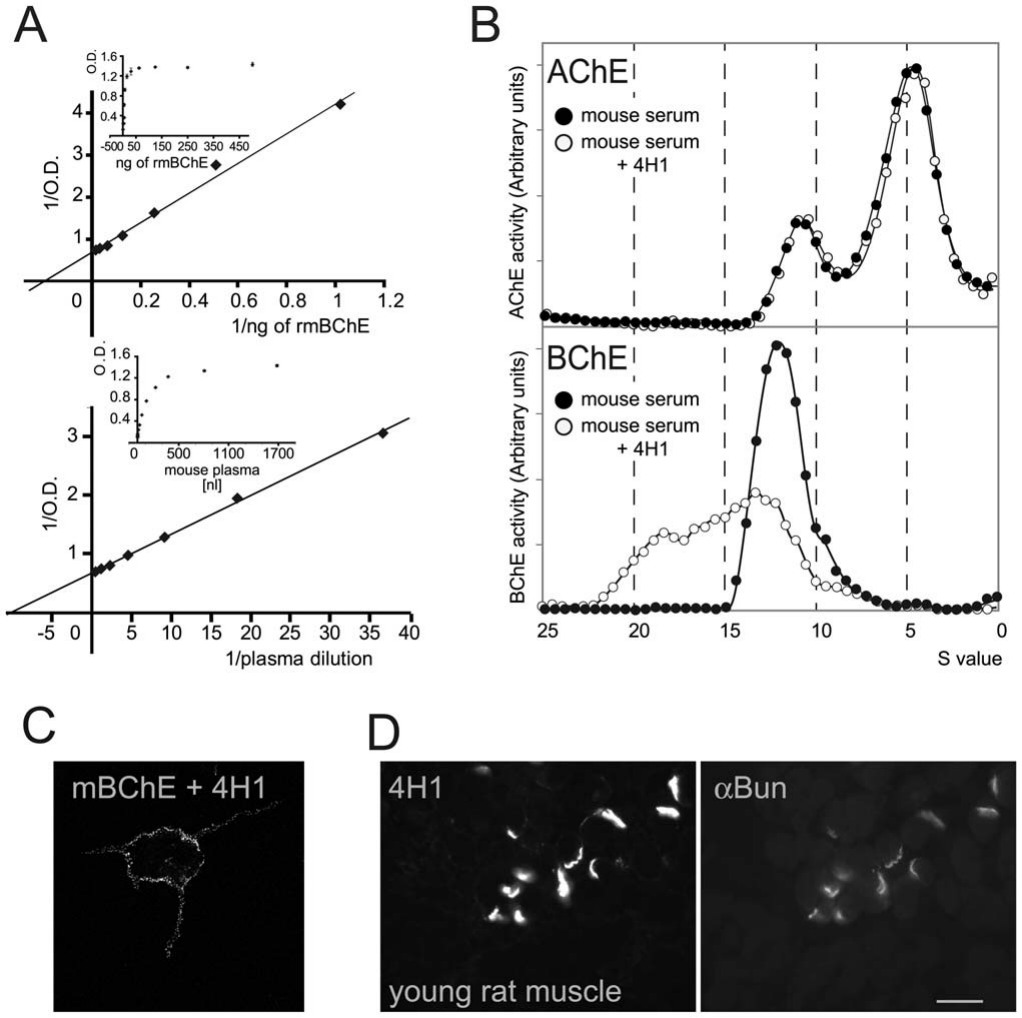
Rodent BChE is recognized by monoclonal antibody 4H1. (A) Selectivity of 4H1 for recombinant and wild-type mouse BChE by ELISA (60 min detection). (B) 4H1 shifts mouse BChE in sucrose gradient. Sucrose gradient of mouse serum. Binding of 4H1 shifts the peaks of tetramer. Wider high-S peak suggests a mixed population of IgG/one tetramer and IgG/two tetramers. (C) 4H1 recognizes mouse BChE expressed by COS cells, anchored PRiMA as documented by confocal microscopy. (D) Immunohistochemistry of young rat neuromuscular junction (NMJ). Right, nicotinic acetylcholine receptors are visualized with bungarotoxin (Bung). Left, 4H1 labels rat BChE.

To evaluate the feasibility of generating an antibody against autologous BChE, we immunized mouse BChE knockouts with mouse BChE protein. Similar to results with immunization of these mice with human BChE, a strong immune response to the antigen was generated and the resultant serum recognized the protein in all assays (figure 4). The antibody titer in the selected mouse sera was more than 1/50,000. Out of 1000 hybridoma, 42 were positive with mouse serum and after two further subcultures three IgG1 were selected, while 4H1 gave the strongest signal. As illustrated in figure 5A, 4H1 detected recombinant mouse BChE and BChE in mouse serum in ELISA. 4H1 shifted BChE oligomers but not AChE oligomers in sucrose gradient (figure 5B). 4H1 binds fixed mouse BChE in transfected mammalian cells (figure 2C) and rat tissue sections (figure 2D). Of note then is that this strategy for antibody generation is not limited to immunization with the orthologous protein, but is efficient even when an autologous protein is used.

## Discussion

Our results show that immunization of knock-out mouse is very efficient method for the generation of both polyclonal and monoclonal antibodies, while both recombinant and natural protein suit the task and antigen preparation determines the characteristic of the resulting antibody. The autologous immunization of knock-out strains of mice has been proposed previously for the generation of antibodies. With this strategy monoclonal antibodies against the prion protein (the infective agent of scrapie) was possible [12]. This method was also successfully used to make a monoclonal antibody against apolipoproteins [13], while another group claimed successful generation of polyclonal but failed in generation of monoclonal antibody against cytokines [14]. We have also successfully used the described strategy to obtain an antibody against another related protein – mouse acetylcholinesterase (data not shown). At present we see no reason why this strategy should not be employed for any protein antigen for which there is need for an antibody, and are purified protein and a cognate knockout mouse strain available. First of all, the number of mutant mice generated over the past 10 years or so has increased dramatically, so much so that several high throughput methods are in progress that will generate a null allele for every single gene (http://www.knockoutmouse.org/ and http://www.eucomm.org/). Importantly, many of these mutants are viable and live with no apparent defect in the phenotype until adulthood. Secondly, large amounts of high quality antigens are available, either from individual studies that produced full/partial recombinant proteins for 3D structure analysis, or from an NIH sponsored initiative underway (http://kb.psi-structuralgenomics.org/about/psi.html) to produce and determine the structure of all major classes of known proteins.

Despite the satisfying results that we have obtained, we would like to point out that the proposed strategy does share some of the limitations of conventional strategies. As with any immunization based strategy, immunoreactivity towards the introduced protein is key but sometimes, for still unexplained reasons, this does not easily occur. Likewise, amounts of the immunizing protein to be used as an antigen need to be available. Moreover, antibodies that are produced may have limited use in recognition of the wild-type protein. The immune response, however, can be different between individual mice making it possible to select the mouse that produces antibodies with favorable properties. Lastly, the antibody could be produced against an epitope that is conserved between species and thus will be cross reactive.

In some selective cases the properties of knockout mouse themselves could be a limiting factor, i.e. because of an impaired immune system or in the case of a partial deletion of the coding sequence, either alternative promoter or splice variants may produce a truncated form of the protein rendering the animal tolerant toward the immunogen. Overall though we feel that the strategy presented, of using knockout mice to produce antibodies against autologous antigens, is a promising method that should be more seriously considered for “problem” antigens, especially in the current climate of new knockout strains emerging seemingly everyday.

## Materials and Methods

### Animals

BChE heterozygous mice [9] were maintained in a mixed background strain 129S1/SvImJ and B6D2. By unsystematic crossing with B6D2 mouse strains, heterogeneity of the background strain was managed in order to avoid random mutations. Homozygous BChE −/− were selected by PCR from crude tissue extracts after alkaline hydrolysis using a mix of allele specific + and − primers (supplementary table S1) and HotStart TaqDNA polymerase (Qiagen), with an annealing temperature of 65°C. Mice were maintained under standard conditions at a constant temperature of 22°C with a 12 - hour daylight cycle. Food and water were provided *ad libitum*. In accordance with French legislation, the investigators had valid licenses to perform experiments on live vertebrates delivered by the Direction des Services Vétérinaires (Préfecture de Police, Paris, France). The animal house and the experimental room had received the agreement of the same authority.

### Immunization

Young (25–40 days old) and old (around 95 days) BChE −/− mice were immunized with 15–50 µg of antigen in Freund's adjuvants (Pierce; # 77140 and #77145) by subcutaneous injection. Mice were bled from femoral vein 5 days after each injection and presence of the antibody in the plasma was tested in ELISA (see below). Once plateau of antibody production was detected, hybridoma fusion was performed (figure 1). Antibody titer was determined by ELISA. As a negative control, four wild-type BChE mice of the same strain and of the same age were immunized under same condition as BChE −/− mice.

### Monoclonal antibody generation and screening

Monoclonal antibodies were generated commercially (P.A.R.I.S Production d'Anticorps & Services, Compiègne, France) by hybridoma fusion (SP2/0) followed by 2 subcloning steps. Hybridomas obtained during preparation were screened for antibody production by modified ELISA method and then by Western blot, immunohistochemistry, by ELISA with human, mouse, rat and cat plasma and mouse tissue extracts (liver, lungs, muscle).

### Isotyping

Isotyping was performed commercially (P.A.R.I.S Production d'Anticorps & Services, Compiègne, France) using an RD Biotech kit. Samples were diluted 1∶10 and color development time was 10 min (sample 11D8; 4H1).

### ELISA

96-well plate Nunc-Immuno F96 Maxi-Sorp plates (Nunc GmbH & Co) were coated with 1 µg of native or denatured recombinant human or mouse BChE per well. Protein was denatured by 20 min incubation at 99°C, in the presence of 1% SDS and 35 mM beta-mercaptoethanol. Plates were blocked with 3% non-fat milk and incubated for 2 hours with 1∶50 and 1∶100 dilution of plasma from immunized animals or with culture medium from hybridoma cells. Secondary anti-mouse antibody coupled to horse radish peroxidase (HRP) (Amersham Biosciences - GE Healthcare Europe GmbH; # NA931) were used in dilution 1∶2000 in phosphate-buffered saline (PBS). HRP was revealed with substrate o-phenylenediamine dihydrochloride (Sigma-Aldrich Chimie; # P5412).

### Modified ELISA

Immunoplates Maxi-Sorp (see above) were coated with 1 µg per well of affinity pure goat anti-mouse IgG +IgM (Jackson Immunoresearch laboratories, # 115-005-004) or goat anti-mouse IgG (P.A.R.I.S Production d'Anticorps & Services, Compiègne, France) diluted into 100 µl with PBS. Plates were blocked with 0.1% BSA. Culture medium from hybridoma cells were incubated in plate, together with or followed by BChE sample. Recombinant human BChE, human plasma, mouse plasma, rat plasma, cat plasma, mouse tissue extracts (liver, lungs, muscle) were used in the assay. Amount of BChE to be used was determined from titration experiments with recombinant human BChE as discussed in supplementary figure S1. Signal was revealed as BChE activity measured with 1 mM butyrylthiocholine, in the presence of 5 mM 5,5′-dithiobis-(2-nitrobenzoic acid) (Ellman's reagent) and 5 mM Hepes buffer, pH 8.0 for the time period up to 20 hours. Sensitivity of the antibody was set to the dilution that gave 40% higher signal comparing to the background.

### Transfection of COS cells

DNA of human BChE in pCR/CMV expression vector was a gift from Dr. David Lenz. Full length human BChE with mutation of four glycosylation sites Asn17, Asn455, Asn481 and Asn486 was in expression vector pGS [10] and DNA of mouse BChE in pGS expression vector were a gift of Oksana Lockridge. rat ColQ [15] and PRiMA [16] were in pCDNA3. ColQ and PRiMA are a specific collagen and a small transmembrane protein that organize BChE in tetramers and anchor them in the extracellular matrix (ColQ) [15] or in the plasma membrane (PRiMA) [16]. To address BChE to the cells surface, COS cells were co-transfected with DNAs of BChE and ColQ or PRiMA. ExGene 500 (Euromedex; # ET0250) was used for transfection. Cells were grown on poly-lysine coated glass slides.

### Immunohistochemistry of transfected COS cells

BChE was detected at the light microscopic level by immunofluorescence. In brief, cells on glass slides were fixed in 2% paraformaldehyde in PBS, washed, incubated in 0.1% BSA for 30 min and then in different dilutions of serum from immunized mice or in different dilutions of culture medium from cells producing anti-BChE antibody with 0.1% BSA for 15 hours at room temperature. After a second wash, slides were incubated with cyanine 3-conjugated donkey anti-mouse. After washing, the cells were mounted in Vectashield mounting medium (Vector Laboratories, Burlingame, CA; H-1000) and examined in a confocal microscope.

### Western Blot

Denatured and reduced purified human BChE were run on NuPAGE Novex 10% Bis-Tris gel (Invitrogen; # NP0303BOX). Denatured and non-reduced plasma samples (maximum load 2 µl per well) were run on NuPAGE Novex 4–12% Bis-Tris gel (Invitrogen, # NP0323BOX). Monomer of BChE runs close to albumin on PAGE and the thick band of albumin in plasma resulting from its high quantity blocks interaction of antibody with BChE. Using denaturing but non-reducing conditions overcomes this problem and allows good separation of albumin and BChE (which remains in dimers). Protein was transferred to PVDF membrane by iBlot Dry Blotting system (Invitrogen, # IB4010) and protein transferred was confirmed by visualization with Ponceau. Membrane was then blocked with 5% non-fat milk and incubated with anti-BChE antibody containing sample. Secondary anti-mouse antibody coupled to HRP (Amersham Biosciences - GE Healthcare Europe GmbH; # NA931) were used in dilution 1∶10000 in PBS. ECL Western Blotting detection reagent (Amersham Biosciences - GE Healthcare Europe GmbH, # RPN2106) was used to detected the signal.

### Sucrose gradient

Transfected cells were extracted in 25 mM Tris-HCl pH 7.4, 0.8 M NaCl, 10 mM EDTA, 1% CHAPS and protease inhibitors 2 mM benzamidine and mouse serum was diluted by 2 in the same buffer. The extracts were incubated with 1 µg of mABs on ice for few hours. Sedimentation analyses of BChE molecular forms were performed in 5–20% (wt/vol) sucrose gradients containing the same buffer except CHAPS was replaced by 0.2% Brij-97 (polyoxyethylene 10 oleoyl ether, SIGMA Aldrich), to shift amphiphilic oligomers. The gradients were centrifuged at 38,000 rpm at 7°C for 18.5 h, using a SW41 rotor (Beckman Instruments). Each gradient was collected in 48 fractions and assayed for BChE activity. Fractions were calibrated with internal sedimentation markers (alkaline phosphatase (6.1S) and ß-galactosidase (16S)). Sedimentation marker profiles were used to establish a linear relation between fraction numbers and Svedberg units.

## Supporting Information

Figure S1 Titration of 11D8 by ELISA. 11D8 was titrated in a plate coated with secondary antibody (1 µg/well). Based on the preliminary studies with polyclonal anti-hBChE antibody (intermediate product), 0.1 µg of rhBChE (70 mU) was used for titration. Amount of bound BChE was determined by an activity assay with butyrylthiocholine (1 mM) in the presence of DTNB. Hydrolysis was followed over a period of up to 4 hours, at variable intervals. Figure represents results obtained after 15 min of development.
(0.17 MB TIF)

Table S1 Primers designed for genotyping of heterozygous and homozygous BChE mutant mice.
(0.03 MB DOC)
