# Supplementary figures and images for: A Novel System for the Efficient Generation of Antibodies Following Immunization of Unique Knockout Mouse Strains
Source: PLoS One. 2010 Sep 23;5(9):e12892. doi: 10.1371/journal.pone.0012892 (PMC2944837; doi:10.1371/journal.pone.0012892)

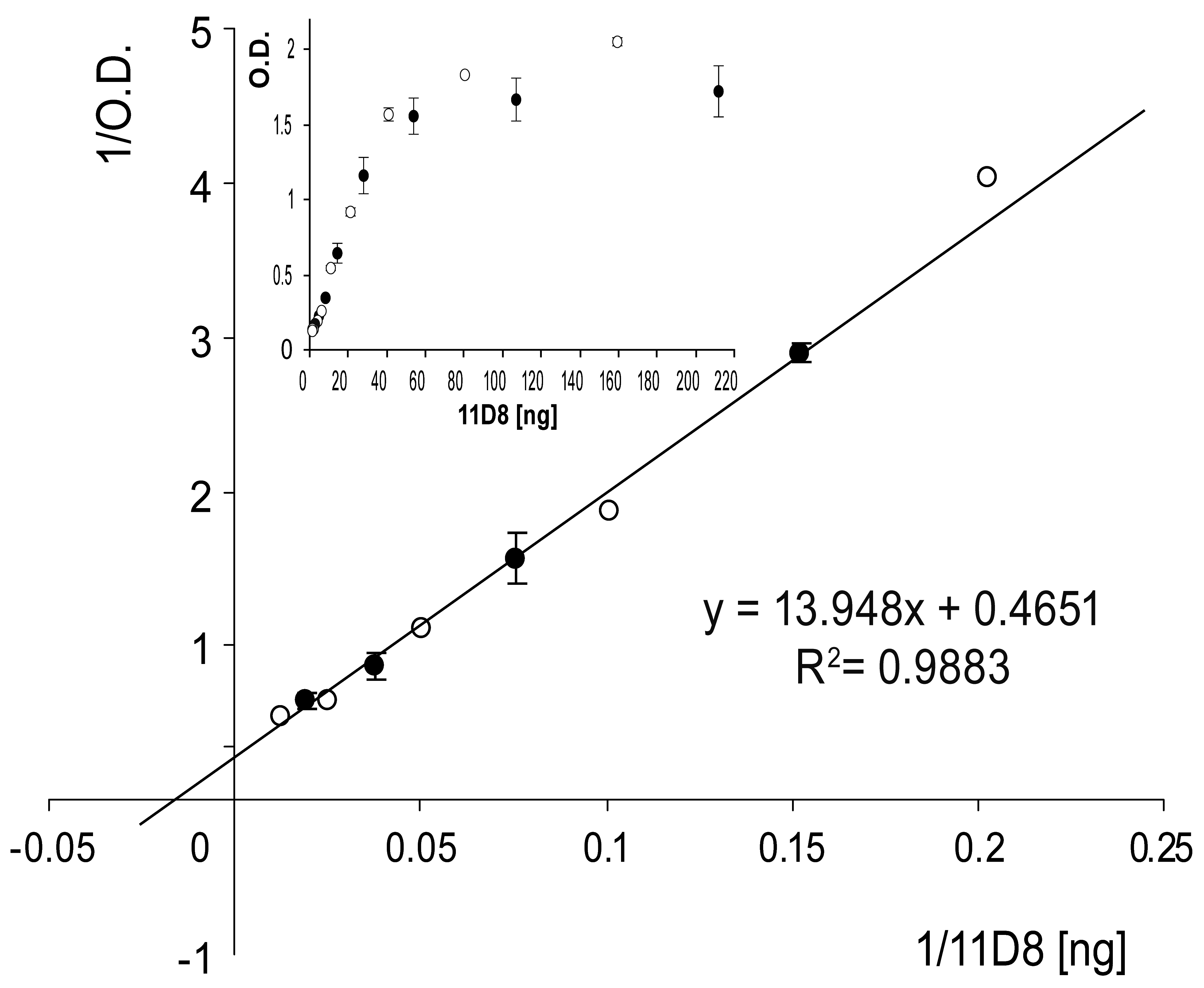

Supplement: Figure S1 — Titration of 11D8 by ELISA. 11D8 was titrated in a plate coated with secondary antibody (1 µg/well). Based on the preliminary studies with polyclonal anti-hBChE antibody (intermediate product), 0.1 µg of rhBChE (70 mU) was used for titration. Amount of bound BChE was determined by an activity assay with butyrylthiocholine (1 mM) in the presence of DTNB. Hydrolysis was followed over a period of up to 4 hours, at variable intervals. Figure represents results obtained after 15 min of development. (0.17 MB TIF) [file pone.0012892.s001.tif]
