# Supplementary material for: A Novel System for the Efficient Generation of Antibodies Following Immunization of Unique Knockout Mouse Strains
Source: PLoS One. 2010 Sep 23;5(9):e12892. doi: 10.1371/journal.pone.0012892 (PMC2944837; doi:10.1371/journal.pone.0012892)
Supplement: Table S1 — Primers designed for genotyping of heterozygous and homozygous BChE mutant mice. (0.03 MB DOC) [file pone.0012892.s002.doc]

| **BChE** | **primers** | **sequence** | **size amplicon** |
| --- | --- | --- | --- |
| **allele +** | **BCHE sens wt** | ATCCATAATGCCACTCAATATGCA | 180 bp |
|  | **BCHE rev** | ATAGATCCATACCATGACAGTGGC |  |
| **allele -** | **BCHE sens mut** | TCTAGTTGCCAGCCATCTGTTGTT | 412 bp |
|  | **BCHE rev** | ATAGATCCATACCATGACAGTGGC |  |
